# Palliative care in Malawi: a scoping review

**DOI:** 10.1186/s12904-023-01264-8

**Published:** 2023-10-04

**Authors:** Natalie Palumbo, Alyssa Tilly, Eve Namisango, Christian Ntizimira, Lameck Thambo, Maria Chikasema, Gary Rodin

**Affiliations:** 1 Schulich School of Medicine and Dentistry, Western University, 1151 Richmond St, London, ON Canada; 2 Division of General Medicine and Clinical Epidemiology and Palliative Care Program, University of North Carolina at Chapel Hill, Chapel Hill, NC USA; 3 African Palliative Care Association, Kampala, Uganda; 4 Cicely Saunders Institute, King’s College London, London, UK; 5 African Center for Research on End of Life Care, Kigali, Rwanda; 6 Palliative Care Association of Malawi, Lilongwe, Malawi; 7 UNC Project-Malawi, Lilongwe, Malawi; 8 Department of Supportive Care, Princess Margaret Hospital, University Health Network, Toronto, ON Canada; 9 Princess Margaret Hospital, University Health Network, Global Institute of Psychosocial, Palliative and End-of-Life Care (GIPPEC), University of Toronto, Toronto, ON Canada; 10 Department of Psychiatry, University of Toronto, Toronto, ON Canada

**Keywords:** Palliative care development, Malawi, Public health

## Abstract

**Background:**

Universal access to palliative care remains a distant goal in many low resource settings, despite the growing evidence of its benefits. The unmet need for palliative care is evident in Africa, but great strides in palliative care development have occurred in several African countries. Located in sub-Saharan Africa, Malawi has been regarded as an exemplar of progress in this area that is achievable in a low resource region. This scoping review examined the literature on the development and state of palliative care in Malawi according to the pillars of health care policy, medicine availability, education, implementation, research activity, and vitality of professionals and advocates.

**Methods:**

A scoping review was conducted of the MEDLINE, Embase, Global Health, CINAHL, Web of Science and PsycINFO databases, as well as grey literature sources. Articles were included if they explored any aspect of palliative care in Malawi.

**Results:**

114 articles were identified that met the inclusion criteria. This literature shows that Malawi has implemented diverse strategies across all pillars to develop palliative care. These strategies include creating a national stand-alone palliative care policy; integrating palliative care into the curricula of healthcare professionals and developing training for diverse service providers; establishing systems for the procurement and distribution of opioids; implementing diverse models of palliative care service delivery; and launching a national palliative care association. Malawi has also generated local evidence to inform palliative care, but several research gaps were identified.

**Conclusions:**

Malawi has made considerable progress in palliative care development, although initiatives are needed to improve medicine availability, access in rural areas, and socioeconomic support for patients and their families living with advanced disease. Culturally sensitive research is needed regarding the quality of palliative care and the impact of therapeutic interventions.

**Supplementary Information:**

The online version contains supplementary material available at 10.1186/s12904-023-01264-8.

## Introduction

The World Health Organization (WHO) defines palliative care as an approach to improve the quality of life of patients and families facing the challenges associated with life-threatening illness [1]. Although palliative care has been linked to the care of patients with advanced cancer, there is increasing evidence for its value in non-cancer populations [2]. However, universal access to palliative care remains a distant goal, particularly in low resource regions.

Of the 40 million people in the world who require palliative care, almost eighty per cent live in low- and middle-income countries, where access is often extremely limited or non-existent [1, 3]. This unmet need is particularly evident in Africa, due to the high prevalence of life-limiting conditions on this continent. In 2020, there were more than one million cases of cancer in Africa and more than 700,000 cancer-related deaths [4], with exponential growth in cases and mortality expected in the coming decades [5]. It is predicted that by 2060 Africa will have the second greatest proportional regional rise in serious health-related suffering [6].

Although access to palliative care remains limited in many parts of Africa [7], there may be important lessons to be learned from countries in this region where progress has been made in its development and integration into health care [7, 8]. One such country is Malawi, a sub-Saharan country, with a population of more than nineteen million people [9]. We report here the findings from a scoping review of palliative care development in Malawi, aiming to identify potentially generalizable strategies that facilitated this progress. These will be considered in relation to the four-pillars of the public health strategy recommended by the WHO to improve palliative care access [10] and endorsed by the African Palliative Care Association for their relevance in Africa [11]. These pillars are health care policy, medicine availability, education, and implementation [10]. Research activity is also considered as a fifth pillar in this scoping review as locally-relevant, high-quality evidence is needed to underpin the four WHO pillars and improve clinical practice [12, 13]. Finally, vitality of professionals and advocates has been recognized as an important driver of palliative care development and is considered in the review [7].

### The context of Malawi

Malawi is a landlocked country located in southeastern Africa that is bordered by Mozambique, Zambia and Tanzania. Malawi is ranked as the sixth poorest country in the world, with more than two-thirds of the population living below the international poverty line [14]. There is a heavy burden of disease in this country, with a high prevalence of malaria and HIV/AIDS [15]. The incidence and mortality of noncommunicable diseases, particularly cancer, also continues to rise [16, 17]. Health care services are provided by the public, private for profit, and private not for profit sectors [18]. Within the public sector, the provision of health care is free and organized into primary, secondary and tertiary levels [18]. However, catastrophic out-of-pocket expenditure is common following the diagnosis of advanced cancer [19].

In the 2015 Quality of Death (QOD) Index, Malawi was ranked 66th of 80 countries in the quality of palliative care [20]. This rank is only slightly lower than that of Kenya, a country that is considered to be a leader in palliative care development in Africa [8]. Since the publication of the QOD Index, Malawi has been recognized as having achieved advanced integration of palliative care services into mainstream health care provision [21], making it the only low-income country in the world to achieve this categorisation [22]. Thus, Malawi may be regarded as an exemplar of progress in palliative care that is achievable in a low resource region.

## Methods

A scoping review was conducted to map the existing literature on palliative care in Malawi. The framework for this review was based on the methodology proposed by Arksey and O’Malley [23] and enhanced by Levac and colleagues [24]. The scoping review approach was chosen because it is well-suited for reviewing a broad range of literature and identifying research gaps.

### Research question

The overall research question was: What is known about palliative care in Malawi, based on the published literature? Sub-questions were: What strategies have been used to promote palliative care development in Malawi? What are the gaps in palliative care research in Malawi?

### Search strategy

A three-step search strategy was utilized to identify publications relating to palliative care in Malawi. First, an information specialist from McMaster University helped identify relevant databases, search terms and subject headings. The keywords and subject headings identified from a limited search of MEDLINE informed the full search. Second, an Ovid search of the MEDLINE, Embase and Global Health databases was conducted. The following grey literature sources were searched: the African Palliative Care Association (APCA) and Palliative Care Association of Malawi (PACAM) websites, and the WHO Regional Office for Africa Library. The search of APCA and PACAM was restricted to the resources/publications sections of the websites. Third, the reference lists of included publications were examined for additional articles. The search was initially conducted in February of 2021, followed by an updated search in May 2022. In response to reviewer feedback, the databases CINAHL, Web of Science and PsycINFO were also searched up to May 2022. There was no publication date or language restriction. A review protocol was not published.

The search strategy consisted of two concepts: palliative care and Malawi. Terms for palliative care such as “palliat*,” “hospice,” and “end-of-life” were included. Terms for Malawi included “Malawi,” “Sub-Saharan Africa,” and “Southern Africa”. These two concepts were combined using the AND operator (see Table 1, supplementary material). Keywords and subject headings related to the broader region of Africa were included to maximize the sensitivity of the search.

### Study selection

Articles retrieved from MEDLINE, Embase, Global Health, CINAHL, Web of Science and PsycINFO were uploaded to Covidence for deduplication and screening. Each article was independently reviewed by two reviewers at the title/abstract level to determine whether it satisfied the inclusion criteria (options: yes, no, and maybe). Articles in the “maybe” category proceeded to full-text review by both reviewers. Any discrepancies in the reviewers’ responses during title/abstract and full-text screening were solved by the decision of a third senior reviewer.

### Inclusion/Exclusion criteria

Articles were included if they explored any aspect of palliative care in Malawi. There was no restriction on publication type. Articles were excluded if palliative care was not the main subject or a specific topic, or if they had limited or no information regarding Malawi. Abstract posters of included studies were excluded.

### Charting the data

Data from eligible articles was collected in an electronic spreadsheet. The following information (if applicable) was extracted from each article: author(s), year of publication; type of publication; pillar(s) of palliative care addressed; details of strategy; study type; study location; study aim(s); study population and sample characteristics; methodology/methods; key findings and conclusion.

### Collating and summarizing the results

The narrative account of the studies was presented in two ways. First, a basic numerical description of the type of articles was included. For research papers, the kind of study was also quantified, which highlighted the dominant areas of research focus and methods. Second, the extracted data were organized into the pre-existing themes of the pillars of palliative care development. This process involved coding the extracted data according to the different elements of the pillars. This approach allowed for a comprehensive exploration of the topic, ensuring that the review addressed diverse dimensions of palliative care development. The methodology is similar to that employed by Rhee et al. in their scoping review published in Lancet Oncology [7].

## Results

### Search results

**Fig. 1:**
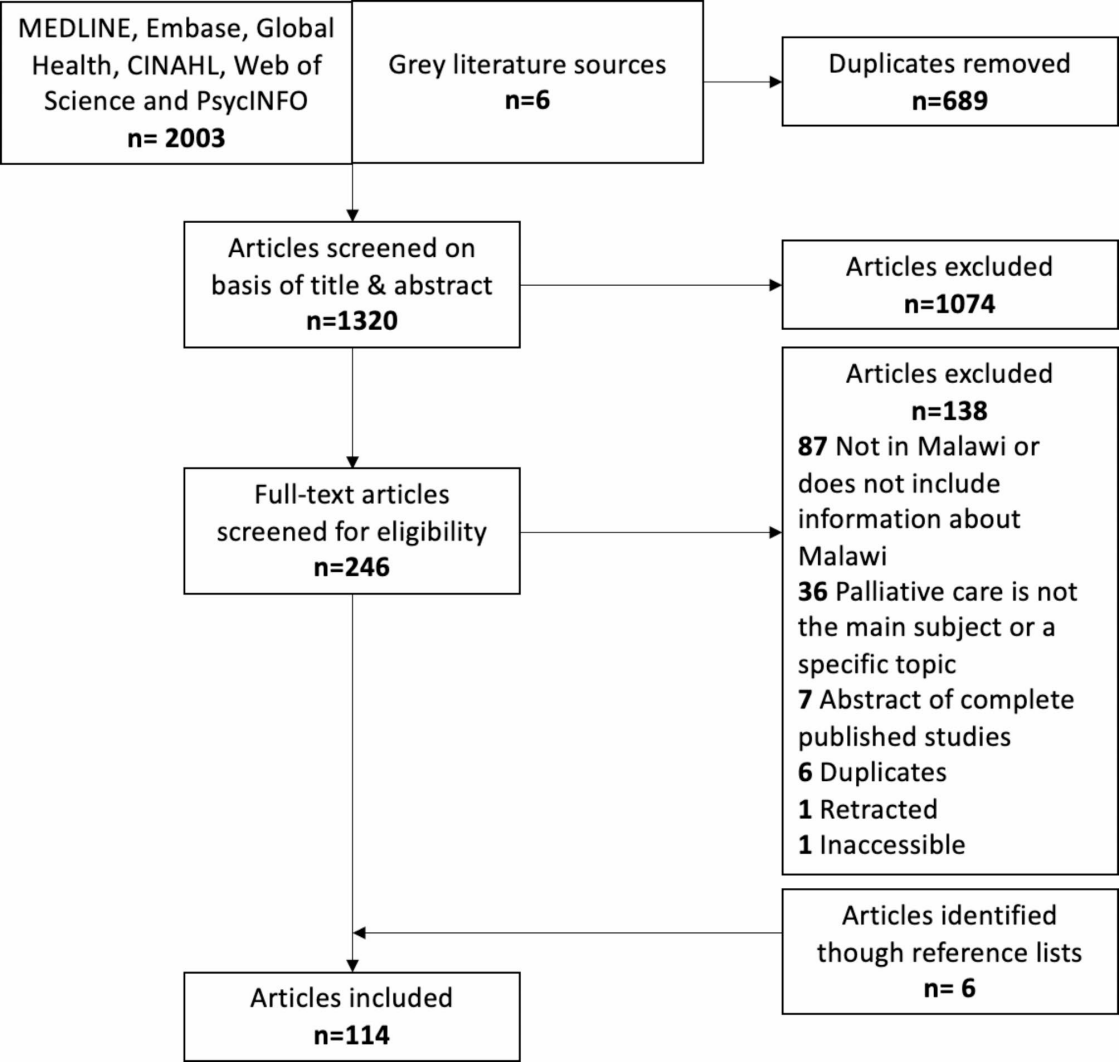
Flow diagram showing each stage of the scoping search process

From the database searches, 2003 articles were identified. Searches of grey literature sources located an additional six articles. The de-duplication process by Covidence removed 689 duplicates, leaving 1314 articles for screening. After excluding those that did not meet the inclusion criteria, 108 articles remained. An additional six relevant articles were identified from the reference lists of included studies. Thus, a total of 114 articles were included in this review (Fig. 1).

The publication types of included articles were: quantitative studies (n = 27), qualitative studies (n = 10), mixed-method studies (n = 5), rapid evaluation field studies (n = 2), reviews (n = 22), conference abstracts (n = 13), reports (n = 10), commentaries (n = 11), study protocols (n = 2), and other (n = 12). Articles in the ‘other’ category were two correspondences, a Lancet news article, four viewpoint articles, an interview transcript, a briefing paper, a blog post, a perspective paper, and a policy document. All articles were published in English between 2003 and 2022. Of the articles published in journals, 90 (84%) were published in non-African journals, and 17 (16%) were published in African journals.

### Palliative Care Development in Malawi

#### Palliative Care Education and Training

Palliative care educational and training interventions have been implemented at multiple levels in Malawi. The University of Malawi College of Medicine, the only medical school in Malawi, incorporated examinable palliative care modules in the second to fifth (final) year of training [25]. Pediatric palliative care is included in the curriculum of pediatric training in the third and fifth year [26]. At the postgraduate level, palliative care training is a part of the Family Medicine training program [22]. Palliative care information is also included in the undergraduate nursing curricula in Malawi [7, 27], although nursing schools do not yet have cancer or palliative care specialties [28]. Previous to the introduction of a Bachelor of Science Degree in Palliative Care in 2018 [25], those in nursing who wished to specialize in palliative care typically left the country for training, which could be economically difficult [28].

The palliative care knowledge base of diverse care providers has been advanced through a variety of courses in Malawi. In 2006, the Ministry of Health and PACAM launched a five-day “Introduction to Palliative Care” course for health care workers [29]. It has become the most common mode of palliative care training outside the clinical learning environment, and has been attended by paralegal, spiritual and social workers [25]. The course is accredited and certified by the Ministry of Health and, as of 2016, was the only accredited palliative care training course in Africa [30]. There is a two-week supplement to the course, but access has been limited by funding and human resource constraints [25]. To improve access to pediatric palliative care education, experts from Botswana, Malawi and Uganda developed an online pediatric palliative care course, which has been completed by nursing/medical students and healthcare professionals in Malawi [31].

Partnerships with international academics, professionals and institutions have helped to strengthen palliative care education and training in Malawi [32–36]. For example, an international palliative care physician-educator provides longitudinal training and clinical mentorship to clinical providers in the Neno Palliative Care Program [32]. Additionally, an international expert from Kenya visited Malawi to train physicians and nurses from across the country in the use of self-expanding metal stents as a palliative treatment [35]. International partnerships with the University of Alabama at Birmingham School of Nursing have also strengthened the oncology nursing education in Malawi [28, 33].

Two additional opportunities were recently introduced to advance education and training in palliative care in Malawi. Ndi Moyo Palliative Care in Salima district launched a five-week Palliative Care Initiators’ Course for clinicians and nurses, and The University of Malawi College of Medicine introduced a three-year Bachelor of Science Degree in Palliative Care that welcomed its first cohort of students in 2018 [22, 25]. This Bachelor program is intended for clinical officers and nurses and is one of the few degree courses in palliative care in Africa [25].

#### Policy

Several policy initiatives and frameworks have guided palliative care development in Malawi. In 2002, Malawi introduced a palliative care approach for the care of people living with HIV/AIDS [37]. Malawi has a stand-alone national palliative care policy, which was developed in 2014 [7]. Additionally, there is a palliative care component in Malawi’s non-communicable disease national plan [38]. The country also has in place a palliative care monitoring and evaluation system, and has dedicated palliative care funding within its national health budget [30]. In 2017, Malawi integrated palliative care indicators into the District Health Information System (DHIS2) platform, making this data available at the national level [39, 40]. Malawi is one of only two countries in sub-Saharan Africa to integrate palliative care indicators into its national health management information system [39].

There has also been attention in Malawi to the impact of health policy on access to opioids. A study published in 2013, as part of the Global Opioid Policy Initiative project, identified four regulatory barriers to opioid access for pain relief in Malawi. These were prescription duration limited to two weeks, the bureaucratic burden of prescriptions, restricted dispensing sites and negative language in laws [41]. It was found that physicians were unable to prescribe an amount of opioid analgesics sufficient to provide pain relief for more than two weeks at a time [41]. Since that time, Malawi has introduced initiatives to modify such restrictive regulations [30].

#### Medicine availability

Malawi has made considerable progress in expanding access to analgesic medication and in 2018 was ranked third in Africa within the palliative care medicines category [42]. Malawi adopted the WHO List of Essential Medicines, which includes palliative care medications [43]. The Central Medical Store is now responsible for the bulk ordering of essential palliative care medicines [43], and morphine availability is monitored by a national task force led by PACAM [22]. In the public sector, essential opioid formulations are provided free of charge [41, 44].

Broadening the range of opioid prescribers is an important strategy to improve opioid accessibility. Malawi has allowed oncologists, surgeons and family physicians to prescribe opium and, in emergency situations, nurses are also permitted to prescribe opioids [41]. Following Hospice Africa Uganda’s lead, it was informally agreed by the Ministry of Health that opioids could be prescribed by nurses and other clinicians who have received specialized training [45]. However, the Malawi Council of Nurses had not adopted this change as of 2014, leaving nurses unclear of their role in this area [45].

In the late 2000s, PACAM began working with the Pharmacy, Drugs and Poisons Board and the Ministry of Health to request suitable quotas from the International Narcotics Control Board, and to establish systems for procurement, reporting and distribution of opioids [29]. However, of the seven essential opioid formulations, only codeine, immediate release morphine and controlled-release morphine were available on formulary by 2013 [41]. The availability of these opioid formulations for patients with a prescription was inconsistent [41]. Further, a report published by PACAM in 2014 found that there were still challenges with morphine procurement [45]. Independent palliative care centres were required to order morphine through the closest District or Central hospital, where supplies were inconsistently available, and the process of obtaining the appropriate signatures could be lengthy. Consequently, the PACAM report recommended that there should be a universal system of procurement, by which established palliative care providers could order directly from the Central Medical Store [45].

APCA has played an important role in improving access to morphine for patients in Malawi and other African countries [45, 46]. This organization identifies countries with challenges regarding morphine access, and invites key personnel from those countries for experiential visits to Uganda to learn from the best-practices in oral morphine manufacturing, distribution and access [46]. Delegations from Malawi have participated in these experiential learning visits, leading to steps in Malawi to improve access to oral morphine and establish capacity-building activities in this country [46].

As a result of the initiatives that were undertaken to improve opioid availability for palliative care, morphine use roughly doubled in Malawi between 2009 and 2013, from 0.41 to 0.82 mg/per capita [25]. There was also an annual increase in the opioid quota in Malawi, which is as an indicator of the spread of palliative care [45]. However, despite progress in opioid availability, morphine stockouts still occur at hospitals in Malawi [47, 48], especially in district hospitals outside of the larger towns [25]. Additionally, focus group interviews with health care workers in Malawi found underlying fears that opioids encourage addiction and hasten death to be common [45]. Such fears among clinicians about opioid usage have also been found in other studies in Malawi [43, 49], and initiatives to promote attitude changes in this area have been undertaken [30].

#### Implementation

By 2014, Malawi had integrated palliative care into its public health systems [50] and palliative care programs now exist at the primary, secondary and tertiary level of care [51]. There are also diverse models of palliative care service delivery, including home-based, outpatient, and inpatient care [51, 52]. The majority of programs are based in hospitals, but there are some non-governmental, stand-alone palliative centres [25]. Some of these stand-alone centres, such as NdiMoyo Palliative Care Centre, are aligned with government hospitals [16]. Information from the review suggested an absence of dedicated in-patient facilities for palliative care [25].

Home-based palliative care programs are offered by a few hospitals in Malawi and by independent voluntary health organisations [52, 53]. Community health workers (CHWs) provide holistic home-based care, often under the supervision of a nurse, and play an important role in the delivery of palliative care. Studies in Malawi describe CHWs assisting with feeding and physical rehabilitation, providing emotional and spiritual comfort, and making referrals when patients require further medical treatment [52, 54]. Through a network of CHWs that link households with health centers and hospitals, the integrated community-based home care model has improved palliative care access, especially in rural settings [32, 52].

Palliative care clinics within hospitals provide services to inpatients referred from various departments and may also service outpatients [55]. One notable example is Tiyanjane Clinic within Queen Elizabeth Central Hospital (QECH), the largest government tertiary facility in Malawi [55]. Since 2003, Tiyanjane Clinic has provided adult palliative care services through hospital and community-based teams in Blantyre [56]. Tiyanjane Clinic exemplifies how partnerships with multiple stakeholders can improve the delivery of palliative care services [55], and such care can potentially reduce the household costs associated with cancer [19].

Malawi was the first African country to have specialist palliative care services for children [26]. The Umodzi program, based at QECH, is an example of a best practice service model for children’s palliative care in Malawi [57] and demonstrates that comprehensive provision is possible in a low resource setting [58]. A strength of this program is that it is integrated into the paediatric and general health system [57]. The Umodzi team provides care across the hospital and also conducts home visits [26, 57].

Overall, Malawi can be considered to have made substantial progress in palliative care provision for both adults and children. Global mapping of palliative care development shows that Malawi moved from category 3 (generalised provision) in 2006 to category 4a (preliminary integration) in 2011 to category 4b (advanced integration) in 2017 [21, 59]. However, challenges for service provision that remain include the reliance of centres of excellence for palliative care on external donor funding [56] and rural populations having inadequate access [19, 25]. In that regard, a recent study at QECH found that individuals living in extreme poverty in rural areas were less likely to receive palliative care [19], highlighting the need for initiatives to link patients in rural areas to established palliative care programs.

#### Vitality of professionals and advocates

Vitality refers to the measurable presence of a critical mass of advocates and professionals participating in specific palliative care activities and promoting key objectives [60]. National palliative care organizations are considered an important part of vitality in this field [7]. In Malawi, the early development of palliative care was largely due to the efforts of a few dedicated individuals promoting palliative care beyond their own hospitals and offering introductory courses to other professionals [61, 62]. PACAM was later formed in 2005 to lead a national effort to scale up affordable, high-quality and culturally appropriate palliative care in Malawi [30]. PACAM has become a national network of individuals and organizations that supports projects to improve access to palliative care, collaborates with the government to develop policies, and provides palliative care training [26, 29, 40, 55, 63–65].

One of PACAM’s major projects is the STEP-UP programme, funded by the True Colours Trust, which aims to ensure the provision of palliative care services at the district health system level [66]. Through the STEP-UP program, PACAM supported the Ministry of Health to integrate palliative care indicators into the DHIS2 [40]. PACAM also collaborates with international institutions to meet the need for palliative care in Malawi [36, 61, 64, 67].

#### Research activity

Of the 114 articles included in this scoping review, 44 were primary research studies and six were conference abstracts reporting studies.

##### Study design

Among the 50 research publications, 14(28%) were qualitative studies or conference abstracts reporting qualitative research, 29 (58%) were quantitative studies or conference abstracts reporting quantitative research, 5 (10%) were mixed-method studies, and 2 (4%) were rapid evaluation methodology studies. The research designs of the quantitative studies are shown in Table 1.

**Table 1:** Quantitative study types

Study type	Number of studies
Observational studies	
Survey methodology studies	8
Retrospective case note reviews	5
Prospective cohort studies	4
Retrospective cohort studies	2
Retrospective cross-sectional studies	4
Micro-costing analysis	2
Interventional studies	
Randomized controlled trial	1
Controlled trial	1

##### Thematic analysis

A total of 12 thematic groups emerged across the research publications (Table 2).

**Table 2:** Thematic groups in research publications

Theme	Total publications within this theme (n = 50)	Focus of research
Community Health Workers	5	The role and/or training of community health workers involved in delivering palliative care
Palliative Care Patients and Caregivers	11	The needs and/or perspectives of palliative care patients and/or their caregivers
Culture	1	The cultural dimensions of disclosing the diagnosis of a life-threatening illness to children
Technology	2	The use of technology in the provision of palliative care
Evaluation	11	Evaluation of palliative care programs, including service models and/or patient characteristics and symptoms
Surgery	2	Whether the intent of surgery for cancer patients was palliative or curative
Education	1	The effectiveness of an educational intervention to improve pain management
Opioids	4	The availability of essential medicines across several countries, including Malawi, or the perspectives of health care workers on opioids
Regional or global palliative care development	6	Mapping and characterizing the state of palliative care in countries around the world, including Malawi
Symptom management	2	The effectiveness of palliative treatments
Cost/Savings	3	The cost of palliative care treatment, or potential household savings associated with palliative care
Methodological	2	Developing or evaluating palliative care assessment tools, including the APCA Palliative Outcome Scale

Qualitative research highlighted that patients in Malawi often live under poor conditions [68–70]. Food insecurity was shown to be a major challenge [68–72], and poor housing conditions adversely affects the care provided by home-based palliative volunteers [69]. Gender-based violence was also found to be a challenge for palliative care patients and caregivers [68, 70]. Caregivers of palliative care patients reported numerous responsibilities, including assisting with activities of daily living, maintaining household functioning, and providing medicines [56, 68, 70, 71]. Caregivers also provided company and courage to patients [56] and brought patients to the hospital [70]. Caregivers indicated that their unmet needs in such care provision included: training and education about the patient’s condition; resources such as clinical supplies and financial assistance; support from both the community and nurses [73]; and food support [70]. Community care was considered essential to some patients, and entailed community members giving one-off financial support and taking turns visiting patients, as well as the coordination of support by Chiefs [71].

Only a small number of studies provided some insight into the quality of palliative care in Malawi from the patient perspective. Interviews with patients in palliative care at QECH indicated that counselling and medication helped them return to their household roles and work [56]. In other studies, the APCA Palliative Outcome Scale (POS) was used to evaluate palliative care outcomes [32, 74–76]. A study at Neno Palliative Care Centre found that for patients with pain at baseline and complete documentation, there was a small, non-significant decrease in the mean APCA POS pain score at follow-up (3.0 vs. 2.7) [32]. At QECH, the median APCA POS pain score among patients with HIV-related Kaposi Sarcoma showed a trend towards improvement over time [74]. However, Francis and colleagues [74] observed that patients and families struggled to complete the questionnaire, even with assistance. The authors concluded that tools such as the APCA POS require more refinement to be reliable in their population group.

##### Location of research

Single-site studies in Malawi were predominantly conducted at QECH, Blantyre (n = 10), St. Gabriel’s Hospital, Namitete (n = 6) and in Lilongwe (n = 6). Other locations included NdiMoyo Palliative Care, Salima District (n = 2), Kansungu District (n = 1), Neno District (n = 1) and Mzuzu City (n = 1). Multisite studies within Malawi included the areas of Blantyre, Mzuzu City, Lilongwe, Salima, Zomba, and Ekwendeni. Overall, the research studies covered diverse areas, including urban and rural areas across Northern, Central and Southern Malawi.

## Discussion

This scoping review showed that Malawi has made substantial progress in the development of palliative care along the WHO Public Health Model pillars. There are strong similarities between the strategies used in Malawi to develop palliative care and those used in Kenya and Uganda, two other leaders in palliative care development in Africa [8]. As in Uganda and Kenya, Malawi has included palliative care in the curricula of healthcare professionals. A bachelor’s degree program in palliative care was created in Malawi, and a similar degree is offered in Uganda. Notably, Malawi is the only African country to offer an accredited palliative care training course for diverse health care workers.

As in Uganda [8], Malawi has included palliative care in the health care budget, thereby strengthening the integration of palliative care in its national health system. This integration is also enhanced by Malawi’s stand-alone national palliative care policy, which was established even before that in Uganda [7]. Malawi was called a beacon site for the integration of palliative care indicators into its health management information system, which neither Uganda nor Kenya have yet undertaken [39]. This initiative will allow the country to generate meaningful longitudinal data and enhance service delivery [39].

Malawi has established systems for the procurement and distribution of opioids, and adapted strategies similar to those in Kenya and Uganda to improve opioid availability [8]. Like Kenya, Malawi adopted the WHO List of Essential Medicines (including 14 palliative care medicines) and created national palliative care guidelines. The partnerships between the Malawi Ministry of Health and PACAM also improved opioid availability, as did the public-private partnerships in Uganda [8]. Following PACAM’s recommendation for a universal system of morphine procurement, all palliative care service providers currently procure directly from the Central Medical Store (L. Thambo, personal communication, March 4, 2022). However, progress is still required to ensure that opioids are consistently available and accessible in Malawi, and that hospitals do not suffer stockouts. Given that many patients live in rural areas with little access to hospitals, it has been recommended that nurses in home-based palliative care teams be given prescribing power [77]. Although both the Medical Council of Malawi and the Nurses and Midwives Council of Malawi have granted opioid prescribing power to nurses, the Narcotic Act of 1957 still does not allow for this activity by nurses (L. Thambo, personal communication, March 4, 2022). PACAM has advocated for the Malawian government to review the Narcotics Act, but regime changes may have hindered progress.

The findings from this scoping review show that various areas of palliative care research have been explored in Malawi. Several studies have been conducted to understand the perspectives and needs of patients and caregivers, the clinical characteristics of patients presenting to palliative care clinics, and the services provided to them. Quantitative data has mainly been collected by review of patient case notes, although other methods have also been used. There has been research evaluating different palliative care programs with diverse service delivery models. Another key research area has been on the training provided to CHWs who deliver palliative care. These studies have shown that, after a training program, CHW participants showed significantly greater knowledge and competency in the skills taught [53, 78–80]. Additionally, several published studies have examined the availability and accessibility of palliative care medicines in Malawi, although these publications were not Malawi-specific.

### Future research

This scoping review indicates that there is a substantial body of local evidence to inform palliative care in Malawi, but that further research is needed. This might include research on the number and breadth of palliative care services in Malawi, location of death preferences, and cultural factors that may influence expectations and treatment decisions about palliative care. Future research would benefit from greater attention to the voices of community members, in addition to those of caregivers and health care workers. This would allow for a greater understanding of attitudes towards death and dying, resources that are desired and accessed at the end of life, and opportunities and barriers to accessing palliative care. Additionally, further research utilizing patient-reported outcomes is required to determine the extent to which initiatives that have been developed to improve palliative care in Malawi have been effective in relieving the suffering and promoting the wellbeing of patients and families living with advanced disease. While several palliative care programs have been evaluated, further research is needed to assess the effect of different models on patient outcomes and the scalability of successful models. Lastly, high-quality interventional studies are required to improve the evidence-base for palliative care in Malawi. To ensure the rigour of such research, culturally relevant measurement tools must be developed and validated in this setting.

A Lancet Commission recommended that metrics that most matter to patients, such as the quality of care and patient experience, should be considered in evaluating health system quality [81]. In that regard, a study validating a measure of the quality of dying and death in Malawi from the perspective of patients, based on proxy ratings of caregivers, is now underway [82].

### Notable findings and recommendations

A critical finding that emerged from this scoping review is the extent to which poverty in Malawi influences palliative care needs. Across many studies, patients and caregivers described how food insecurity and economic hardship negatively impacted their wellbeing [32, 52, 68–70, 73]. Many patients needed not only pain relief and clinical support, but adequate housing, support with school fees, and nutritious food. The care of such individuals is compromised when caregivers cannot afford the proper clinical supplies or the recommended foods. Holistic palliative care in this context must therefore include attention to food support and other basic socioeconomic necessities. It is noteworthy that several palliative care programs in Malawi have taken steps to provide socioeconomic assistance to patients and their families. One such strategy was referring eligible patients to partner agencies that can provide nutritional support [32]. Palliative care programs in low-resource settings should take steps to partner with agencies that can provide socioeconomic support, including basic income grants, for patients and families and also provide linkages for patients to participate in government development programmes.

The research evaluating palliative care programs provided evidence for successful service delivery models in Malawi. Several studies showed the importance of networks between the home, hospitals and health facilities to ensure the continuity of palliative care. The use of CHWs are critical in this network of care, especially in rural areas, as they directly engage patients in their home and facilitate appropriate referrals. The integrated home-based care model that linked patients to health care workers and the hospital may be an appropriate model to continue to implement and scale-up in Malawi.

### Limitations

The search strategy for this scoping review did not include all grey literature sources that may have contained information related to palliative care in Malawi. For example, hospital and Ministry of Health websites were not included in the search, but may have valuable information. Another limitation is that published articles may not always accurately capture the current state of palliative care. For example, the published literature mentions that Malawi has no dedicated in-patient facilities for palliative care, although St. Gabriel’s Hospital has had a dedicated inpatient palliative care unit (24 hospice beds) for many years [83].

## Conclusion

This first scoping review conducted on palliative care in Malawi shows that diverse strategies have been successfully employed in the development of palliative care in this country. Further steps are needed to improve the availability and accessibility of opioids and coordinate economic and food support for palliative care patients. Important research has been conducted in this setting, but future research would benefit from the growth of the research infrastructure, with greater capacity to conduct interventional studies. Such studies will require the development and local validation of culturally appropriate and relevant outcome measures. Further research is also needed on the quality of palliative care, cultural dimensions of palliative care, place of care preferences, and the relative efficacy of different models of palliative care delivery. However, the success achieved in Malawi in the development of palliative care provides valuable lessons for other resource-constrained countries.

### Electronic supplementary material

Below is the link to the electronic supplementary material.

Supplementary Material 1

## Data Availability

The datasets used and/or analyzed during the study are available from the corresponding author upon reasonable request.
