# Supplementary material for: Palliative care in Malawi: a scoping review
Source: BMC Palliat Care. 2023 Oct 4;22:146. doi: 10.1186/s12904-023-01264-8 (PMC10548577; doi:10.1186/s12904-023-01264-8)
Supplement: Supplementary file 1 — Supplementary Material 1 [file 12904_2023_1264_MOESM1_ESM.docx]

**Supplementary Material:** Full Electronic Search Strategy for at Least One Database

This supplementary file details the full electronic search strategy for one database included in the scoping review entitled “Palliative care in Malawi: a scoping review”.

Table 1. Sample search strategy in Embase database (initially searched on Feb 23, 2021).

| # | Searches | Results |
| --- | --- | --- |
| 1 | exp palliative therapy/ | 115934 |
| 2 | exp palliative nursing/ | 1011 |
| 3 | exp terminal care/ | 72132 |
| 4 | exp hospice care/ | 10844 |
| 5 | exp hospice patient/ or exp terminally ill patient/ | 8838 |
| 6 | exp hospice/ | 13911 |
| 7 | exp hospice nursing/ | 62 |
| 8 | palliat*.mp. | 166210 |
| 9 | “end-of-life”.mp. | 37599 |
| 10 | “terminal care”.mp. | 37789 |
| 11 | “terminal patient”.mp. | 313 |
| 12 | hospice*.mp. | 28425 |
| 13 | terminal* ill*.mp. | 8 |
| 14 | or/1-13 | 233743 |
| 15 | Malawi/ | 8102 |
| 16 | “Africa south of the Sahara”/ | 14998 |
| 17 | subsaharan africa*.mp. | 322 |
| 18 | southern africa*.mp. | 6909 |
| 19 | Malawi.mp. | 9688 |
| 20 | Malawian.mp. | 1693 |
| 21 | sub-saharan africa.mp. | 32064 |
| 22 | or/15-21 | 52486 |
| 23 | 14 and 22 | 403 |
